# Clinical outcomes in *ALK*-rearranged lung adenocarcinomas according to *ALK* fusion variants

**DOI:** 10.1186/s12967-016-1061-z

**Published:** 2016-10-19

**Authors:** Yoon Jin Cha, Hye Ryun Kim, Hyo Sup Shim

**Affiliations:** 1 Department of Pathology, GangNam Severance Hospital, Yonsei University College of Medicine, Seoul, South Korea; 2 Department of Oncology, Yonsei Cancer Center, Yonsei University College of Medicine, Seoul, South Korea; 3 Department of Pathology, Severance Hospital, Yonsei University College of Medicine, 50-1 Yonsei-ro, Seodaemun-gu, Seoul, 03722 South Korea

**Keywords:** Non-small cell lung cancer, Anaplastic lymphoma kinase, *EML4*-*ALK* fusion, Pemetrexed, Anaplastic lymphoma kinase inhibitor

## Abstract

**Background:**

Clinical outcomes of anaplastic lymphoma kinase (*ALK*)-rearranged non-small cell lung cancer according to *ALK* fusion variants are not clear. We aimed to investigate the prevalence of *ALK* fusion variants and to compare clinical outcomes according to *ALK* fusion variants.

**Methods:**

A retrospective analysis was conducted on patients with advanced *ALK*-rearranged adenocarcinoma treated with chemotherapy and ALK inhibitors. *ALK* rearrangement was identified by fluorescence in situ hybridization and confirmed by immunohistochemistry. Peptide nucleic acid-mediated quantitative polymerase chain reaction assays, designed to detect 28 types of echinoderm microtubule-associated protein-like 4 (*EML)*-*ALK* rearrangements, were performed. Clinicopathological analysis and treatment outcomes with platinum-based chemotherapy, pemetrexed therapy, and ALK inhibitors—including crizotinib and ceritinib—were evaluated.

**Results:**

A total of 52 patients with *ALK*-rearranged lung adenocarcinoma were enrolled. *EML4*-*ALK variant 1* (v1) was the most common variant (38.5 %) followed by the non-*EML4* variant (36.5 %), *EML4*-*ALK variant 3a/b* (19.2 %), and *EML4*-*ALK variant 2* (5.8 %). No clinicopathological distinction was found between the different *ALK* fusion variants. Treatment response rates for each therapeutic agent did not differ according to *ALK* fusion variant. However, *EML4* variants, especially v1, showed significantly longer progression-free survival (PFS) on pemetrexed treatment than did non-*EML4* variants (median 31.1 months versus 5.7 months, *P* = 0.003). PFS with platinum-based chemotherapy and ALK inhibitors did not differ according to *ALK* fusion variant. Multivariate survival analysis using Cox’s regression model revealed v1 as the only predictive factor for prolonged PFS on pemetrexed.

**Conclusions:**

Among *ALK* fusion variants, v1 is the most common subtype. It showed superior progression-free survival on pemetrexed than did non-*EML4* variants. No survival difference was demonstrated between variants treated with crizotinib or ceritinib.

**Electronic supplementary material:**

The online version of this article (doi:10.1186/s12967-016-1061-z) contains supplementary material, which is available to authorized users.

## Background

Anaplastic lymphoma kinase (*ALK)* rearrangements, found in approximately 5 % of non-small cell lung cancers (NSCLCs), are relatively rare genetic alterations compared with epidermal growth factor receptor *(EGFR)* or *KRAS* mutations [1]. Soda et al. identified the echinoderm microtubule-associated protein-like 4 (*EML4)*-*ALK* fusion gene, and reported its transforming activity and potential as a therapeutic target in NSCLCs [2]. Subsequently, following reports of dramatic therapeutic effects of crizotinib on *ALK*-rearranged NSCLCs [3, 4], a number of studies on the clinicopathologic characteristics of *ALK*-rearranged NSCLC have been conducted [5–8]. Currently, the fluorescence in situ hybridization (FISH) method is considered the gold standard for establishment of *ALK*-rearrangement positivity. In addition, immunohistochemistry (IHC) for ALK protein is known to have high sensitivity and specificity for recognition of *ALK* rearrangements and is strongly correlated with FISH results [9, 10]. However, FISH and IHC cannot specify the different variants or fusion gene partners of the *ALK* gene, which can be identified by real time-polymerase chain reaction (RT-PCR) or next-generation sequencing technology. Crizotinib is effective for NSCLC patients harboring *ALK* rearrangements (~60 % of patients achieve an objective response) but almost all experience disease progression after 8–11 months [3, 11, 12]. We hypothesized that different *ALK* fusion variants would lead to different treatment responses. In the present study, we investigated the prevalence of *ALK* fusion partners in NSCLCs, and explored whether the efficacy of therapeutic agents differs according to *ALK* fusion variant.

## Methods

A retrospective analysis was conducted on patients with advanced *ALK*-rearranged adenocarcinoma treated with chemotherapy and ALK inhibitors. This retrospective study was approved by the Institutional Review Board of Severance Hospital (No. 4-2015-0926).

### Clinicopathologic analysis

The following clinicopathologic parameters were recorded: age, sex, smoking status [never smokers, former smokers (quit smoking >1 year before diagnosis), and current smokers], pack-year smoking history (defined as the number of cigarette packs smoked per day multiplied by the number of years of smoking), sites of metastasis, and pathological tumor stage at diagnosis. For histological analysis, intra- and/or extracellular mucin, *ALK*-related growth patterns including cribriform and solid signet ring cells, features of nuclei, and psammomatous calcification were examined. All samples were reviewed by experienced pulmonary pathologists (Y.J.C. and H.S.S.). Treatment methods, treatment responses, overall survival, and progression-free survival (PFS) were assessed. Tumor response was determined according to Response Evaluation Criteria in Solid Tumors, version 1.1 [13].

### EGFR and KRAS mutation analysis

To determine the *EGFR* and *KRAS* mutation status, DNA was extracted from formalin-fixed, paraffin-embedded (FFPE) tissues using the DNeasy Isolation Kit (Qiagen, Valencia, CA, USA), according to the manufacturer’s instructions. For the *EGFR* gene, direct DNA sequencing of exons 18–21 was performed using the PNA Clamp™ *EGFR* Mutation Detection Kit (PANAGENE, Daejeon, Korea). For the *KRAS* gene, direct DNA sequencing of codons 12 and 13 was performed. Each tumor was classified as positive or negative for a mutation after comparison with the wild-type gene sequence.

### ALK fluorescence in situ hybridization and immunohistochemistry

To identify *ALK* rearrangements, FISH was performed using a break-apart *ALK* probe (Vysis LSI Dual Color, Break Apart Rearrangement Probe; Abbott Molecular, Abbot Park, IL, USA). *ALK* rearrangement was scored as positive when >15 % of tumor cells displayed split or isolated signals containing a kinase domain. IHC was performed using an ALK antibody (rabbit monoclonal, clone D5F3, Cell Signaling Technology, Danvers, MA, USA) and Ventana automated immunostainer BenchMark XT (Ventana Medical Systems, Tucson, AZ, USA), as previously described [14].

### RNA extraction and cDNA synthesis

Total RNA was extracted using the PureLink™ FFPE Total RNA Isolation Kit (Invitrogen Carlsbad, CA, USA) with the following protocol modifications. The resulting RNA was eluted in 50 µL of elution buffer. The concentration and purity of the extracted RNA were determined by spectrophotometry. The extracted RNA was stored at −80 °C until required. We used 250 ng of total RNA to generate cDNA using the Super Script VILO cDNA synthesis kit (Invitrogen).

### PNA-mediated qPCR assay for EML4-ALK screening and genotyping

*EML4*-*ALK* fusion RNA was detected using the PANA qPCR™ *EML4*-*ALK* fusion gene detection Screening and Genotyping kit (PANAGENE, Daejeon, Korea), designed to detect 28 known *ALK* rearrangements. Screening for and genotyping of 12 *EML4*-*ALK* fusions was performed, including: E6;A19, E6;A20, E6ins33;A20(3ea), E6;ins18A20, E13;A20(5ea), E13;ins69A20(2ea), E20;A20(2ea), E20;ins18A20(2ea), E14ins11;del49A20(2ea), E14;del14A20, E14;del38A20, E2;A20, E2;ins117A20, E17;ins30A20, E17ins61;ins34A20, E17ins65;A20, E17;ins68A20, and E17del58;ins39A20. Reverse transcription and RT-PCR were performed in a CFX96 RT-PCR detection system (BIO-RAD, Foster city, CA, USA) under the following conditions: 2 min at 50 °C, 15 min at 95 °C and 5 cycles of 10 s at 95 °C, 30 s at 58 °C and 45 cycles of 10 s at 95 °C, and 30 s at 58 °C and 15 s at 72 °C. A positive result was defined as a threshold cycle (Ct) value <40, and a positive internal control was defined as a Ct value <36. A result was regarded as invalid if the assays for *EML4*-*ALK* fusion gene and internal control showed simultaneous negative results. When invalid results were obtained, the assay was repeated using the newly synthesized cDNA. The assay result was interpreted as positive for *EML4*-*ALK* according to the manufacturer’s instructions.

### Statistical analysis

Clinicopathologic parameters were compared using the Chi square (for categorical parameters) and Mann–Whitney *U* (for continuous parameters) tests. Survival was evaluated using the Kaplan–Meier method, and statistical differences in survival times were determined using the log-rank test. A Cox proportional hazards model was used to assess risk factors for PFS of each therapeutic agent. Statistical analyses were performed using SPSS 19.0 (SPSS Inc. Chicago, IL, USA), and a *P* value <0.05 was considered significant.

## Results

### Patient selection

Between March 2000 and February 2015, *ALK*-rearrangement was confirmed in 76 patients at our institution; all had adenocarcinomas. Results of RT-PCR analysis for *ALK* fusion partners were available for 52 of the 76 patients. These 52 patients were included: 19 were diagnosed by transbronchial lung biopsy or fiber optic bronchoscopy biopsy of the primary lung tumor, 6 by endobronchial ultrasound lymph node biopsy, 16 by lobectomy or wedge resection of the primary lung tumor, 5 by pleural biopsy, and 5 by biopsy of distant metastatic lesions.

### Clinicopathologic characteristics and ALK fusion variants

**Fig. 1:**
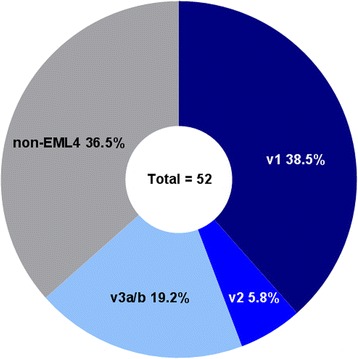
Prevalence of *ALK* fusion variants

Patients’ clinicopathologic characteristics are summarized in Table 1. The median age was 52 (range, 31–76) years and 23 (44.2 %) patients were male. Mean follow-up period was 43.4 ± 7.1 months (range, 2.4–347.0). Thirty-five (67.3 %) were never smokers, 6 (11.5 %) were former smokers, and 11 (21.2 %) were current smokers. In terms of pathologic stage, 5.8 and 94.2 % of cases were stage IIIB and stage IV, respectively, at study start. Twenty-eight (53.8 %) patients had lung or pleural metastasis (M1a), and 35 (67.3 %) had distant metastasis (M1b) (Additional file 1: Table S1). The most common site of distant metastasis was the brain (*N* = 23, 44.2 %). The *EML4*-*ALK variant 1* (v1) was the most common (*N* = 20, 38.5 %), followed by the non-*EML4* variant (*N* = 19, 36.5 %), *EML4*-*ALK variant 3a/b* (v3a/b) (*N* = 10, 19.2 %), and *EML4*-*ALK variant 2* (v2) (*N* = 3, 5.8 %) (Fig. 1).

**Table 1:** Clinicopathologic characteristics and histological analysis

	Total (*N* = 52)	EML4 variants (*N* = 33)	Non-EML4 variants (*N* = 19)	*P* value
Clinicopathologic parameters				
Age, median (range)	52 (31–76)	50 (31–76)	55 (32–70)	0.227
Women (%)	29 (55.8)	21 (63.6)	8 (42.1)	0.132
Smoking history (%) and pack-years				0.904
Never smoker	35 (67.3)	23 (69.7)	12 (63.2)	
Ex-smoker, pack-years	6 (11.5), 17.7	4 (12.1), 19.4	2 (10.5), 14.3	
Current smoker, pack-years	11 (21.2), 23.1	6 (18.2), 19.6	5 (26.3), 27.4	
Pathologic stage				0.546
IIIB	3 (5.8)	1 (3.0)	2 (10.5)	
IV	49 (94.2)	32 (97.0)	17 (89.5)	
Metastasis sites				
Brain	23 (44.2)	14 (42.4)	9 (47.4)	0.730
M1a sites	28 (53.8)	22 (66.7)*	6 (31.6)	*0.015*
M1b sites	35 (67.3)	22 (66.7)	13 (68.4)	1.000
M1ab sites	49 (94.2)	32 (97.0)	17 (89.5)	0.546
Death	19 (36.5)	11 (33.3)	8 (42.1)	0.527
Histologic parameters				
Presence of mucin	10 (22.7)	5 (17.2)	5 (33.3)	0.271
Intracellular, columnar cells	4 (9.1)	1 (3.4)	3 (20.0)	0.107
Intracellular, signet ring cells	7 (15.9)	4 (13.8)	3 (20.0)	0.675
Extracellular	4 (9.1)	1 (3.4)	3 (20.0)	0.107
Predominant pattern				0.443
Acinar	15 (38.5)	8 (33.3)	7 (46.7)	
Solid	18 (46.2)	11 (45.8)	7 (46.7)	
Cribriform	4 (10.3)	4 (16.7)	0 (0.0)	
Micropapillary	2 (5.1)	1 (4.2)	1 (6.7)	
Cribriform pattern	10 (22.7)	7 (24.1)	3 (20.0)	1.000
Solid signet rings	25 (56.8)	17 (58.6)	8 (53.3)	1.000
Prominent nucleoli	17 (38.7)	14 (48.2)	3 (20.0)	0.136
Psammomatous calcification	6 (13.6)	4 (13.8)	2 (13.3)	1.000

* *EML4*-*ALK variant 1* (*N* = 13, 65.0 %); *EML4*-*ALK variant 2* (*N* = 1, 33.3 %); *EML4*-*ALK variant 3a/b* (*N* = 8, 80.0 %)

Among the *ALK* fusion variants, tumors with *EML4*-*ALK* variants showed more frequent lung and/or pleural involvement without distant metastasis compared with the non-*EML4* variants (*P* = 0.015). Among the 22 *EML4* variant tumors with lung and/or pleural involvement, the majority were v1 (*N* = 13, 65.0 % of v1) and v3a/b (*N* = 8, 80.0 % of v3a/b). Other clinicopathologic parameters and histologic features did not differ according to *ALK* fusion variant.

Most patients received platinum-based chemotherapy or pemetrexed therapy as first-line chemotherapy, before receiving crizotinib or ceritinib. Single-agent pemetrexed, *EGFR* tyrosine kinase inhibitors (TKIs), and single-agent platinum therapy were used as second-line or further lines of therapy. Patient treatment history before ALK inhibitor use is summarized in Additional file 1: Table S2.

### Efficacy of chemotherapy regimens and treatment response, according to ALK fusion partners

Forty patients (76.9 %) received first-line platinum-based chemotherapy, with partial response (PR) in 10 (25.0 %), stable disease (SD) in 20 (50.0 %), and progressive disease (PD) in 10 (25.0 %). With regard to pemetrexed, 35 patients (67.3 %) received pemetrexed in any line of treatment: 7 (20.0 %), 25 (71.4 %), and 3 (8.6 %) patients showed PR, SD, and PD, respectively. There were no significant differences in objective response rate (ORR) or disease control rate (DCR) with platinum-based chemotherapy or pemetrexed, according to *ALK* fusion variant.

Overall, 37 patients received ALK inhibitors, including crizotinib (*N* = 32, 61.5 %), ceritinib (*N* = 14, 26.9 %), and alectinib (*N* = 2, 3.8 %). ALK inhibitors were administered as second- or further-line therapy in most patients, except for 8 patients who received crizotinib (*N* = 7) and ceritinib (*N* = 1) as first-line therapy. Five patients (two v1 and three v3a/b), who received crizotinib showed PD while most patients showed at least SD and PR to ALK TKI treatment. Overall, the ORR was 53.1 % with crizotinib and 57.1 % with ceritinib. Treatment response rates to ALK inhibitors did not differ according to *ALK* fusion variant (Table 2).

**Table 2:** Efficacy of ALK inhibitors according to *ALK* fusion variants

	Total (*N* = 52)	*EML4*-*ALK variant 1* (*N* = 20)	*EML4*-*ALK variant 2* (*N* = 3)	*EML4*-*ALK variant 3a/b* (*N* = 10)	Non-EML4 variants (*N* = 19)	*P* value
First-line, platinum-based CTx, *N* (%)	40 (76.9)	17 (85.0)	1 (33.3)	9 (90.0)	13 (68.4)	0.979
PR	10 (25.0)	4 (23.5)	0 (0.0)	3 (33.3)	3 (23.1)	
SD	20 (50.0)	9 (52.9)	1 (100.0)	4 (44.4)	6 (46.2)	
PD	10 (25.0)	4 (23.5)	0 (0.0)	2 (22.2)	4 (30.8)	
ORR, %	25.0	23.5	0.0	33.3	23.1	0.853
DCR, %	75.0	76.5	100.0	77.8	69.2	0.791
Received pemetrexed, any line, *N* (%)	35 (67.3)	17 (85.0)	1 (33.3)	6 (60.0)	11 (57.9)	0.591
PR	7 (20.0)	2 (11.8)	0 (0.0)	3 (50.0)	2 (18.2)	
SD	25 (71.4)	13 (76.5)	1 (100.0)	3 (50.0)	8 (72.7)	
PD	3 (8.6)	2 (11.8)	0 (0.0)	0 (0.0)	1 (9.1)	
ORR, %	20.0	11.8	0.0	50.0	18.2	0.291
DCR, %	91.4	88.2	100.0	100.0	90.9	1.000
Received Crizotinib, *N* (%)	32 (61.5)	10 (50.0)	2 (66.7)	8 (80.0)	12 (63.2)	0.134
PR	17 (53.1)	3 (30.0)	2 (100.0)	4 (50.0)	8 (66.7)	
SD	10 (31.3)	5 (50.0)	0 (0.0)	1 (12.5)	4 (33.3)	
PD	5 (15.6)	2 (20.0)	0 (0.0)	3 (37.5)	0 (0.0)	
ORR, %	53.1	30.0	100.0	50.0	66.7	0.191
DCR, %	84.4	80.0	100.0	62.5	100.0	0.109
Received Ceritinib, *N* (%)	14 (26.9)	5 (25.0)	1 (33.3)	3 (30.0)	5 (26.3)	0.723
CR	1 (7.1)	0 (0.0)	1 (100.0)	0 (0.0)	0 (0.0)	
PR	7 (50.0)	2 (40.0)	0 (0.0)	2 (66.7)	3 (60.0)	
SD	4 (28.6)	2 (40.0)	0 (0.0)	1 (33.3)	1 (20.0)	
PD	1 (7.1)	0 (0.0)	0 (0.0)	0 (0.0)	1 (20.0)	
ORR, %	57.1	50.0	100.0	66.7	62.5	NA
DCR, %	85.7	100.0	100.0	100.0	80.0	NA
Received Alectinib, *N* (%)	2 (3.8)	0 (0.0)	0 (0.0)	1 (10.0)	1 (5.3)	NA
PR	2 (100.0)	0 (0.0)	0 (0.0)	1 (100.0)	1 (100.0)	

*CTx* chemotherapy; *PR* partial response; *SD* stable disease; *PD* progressive disease; *CR* complete response; *ORR* objective response rate; *DCR* disease control rate; *NA* not applicable

A 42 year-old woman, who harbored a v2 variant, showed complete response (CR) to ceritinib. Her brief clinical history and detailed histologic features are summarized in Additional file 1: Fig. S1.

### Progression-free survival with each therapeutic agent, according to ALK fusion variant

**Fig. 2:**
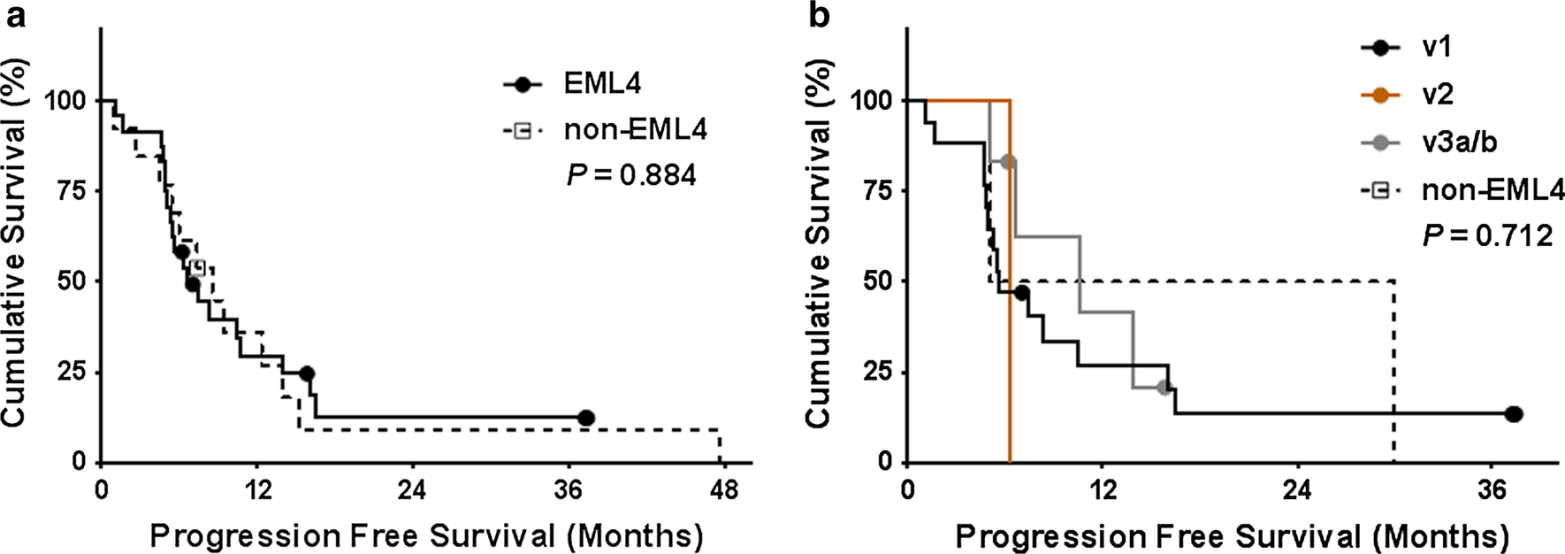
Kaplan–Meier curves of progression free survival of patients treated with platinum-based chemotherapy, according to the *ALK* fusion variants. **a** *EML4* (*N* = 27) versus non-*EML4* (*N* = 13). **b** Demonstration of progression free survival of each variant (v1, *N* = 17; v2, *N* = 1; v3a/b, *N* = 9; non-EML4, *N* = 13). Each symbol on the plot marks a censored patient. v1, *EML4*-*ALK variant 1*; v2, *EML4*-*ALK variant 2*; v3a/b, *EML4*-*ALK variant 3a/b*

**Fig. 3:**
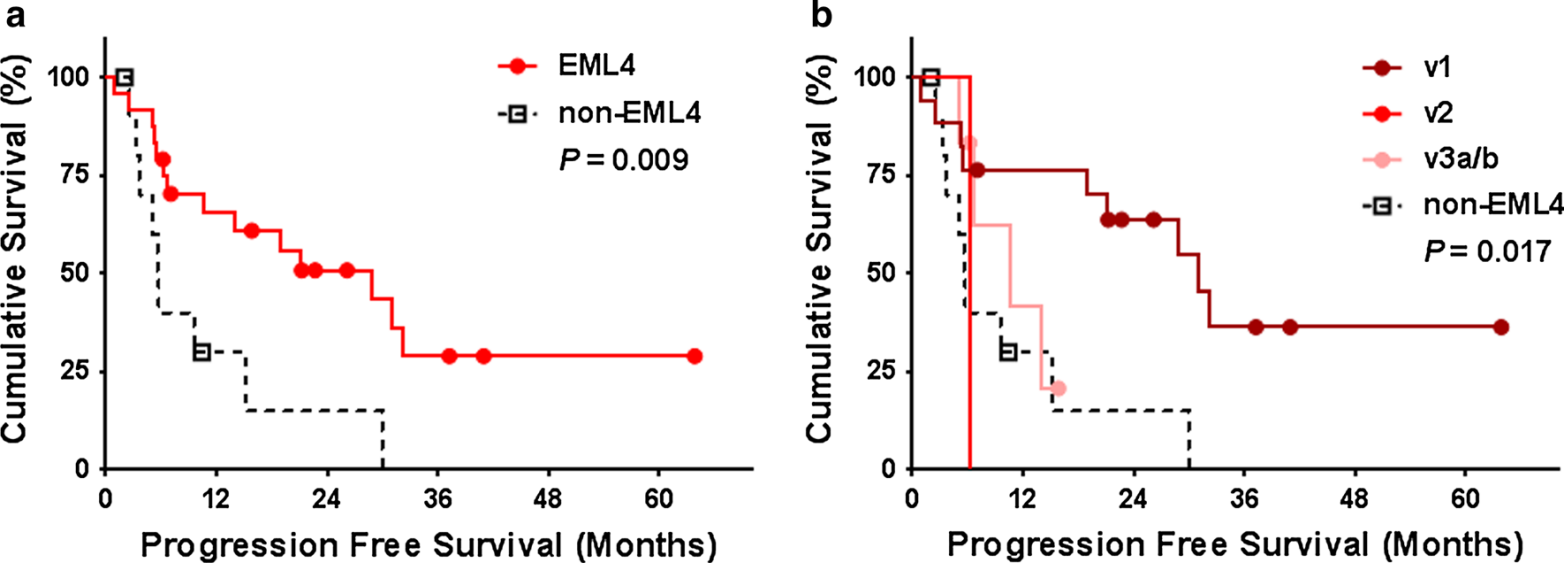
Kaplan–Meier curves of progression free survival of patients treated with pemetrexed as any line, according to the *ALK* fusion variants. **a** *EML4* (*N* = 24) versus non-*EML4* (*N* = 11). **b** Demonstration of progression free survival of each variant (v1, *N* = 17; v2, *N* = 1; v3a/b, *N* = 6; non-EML4, *N* = 11). Each symbol on the plot marks a censored patient. v1, *EML4*-*ALK variant 1*; v2, *EML4*-*ALK variant 2*; v3a/b, *EML4*-*ALK variant 3a/b*

**Fig. 4:**
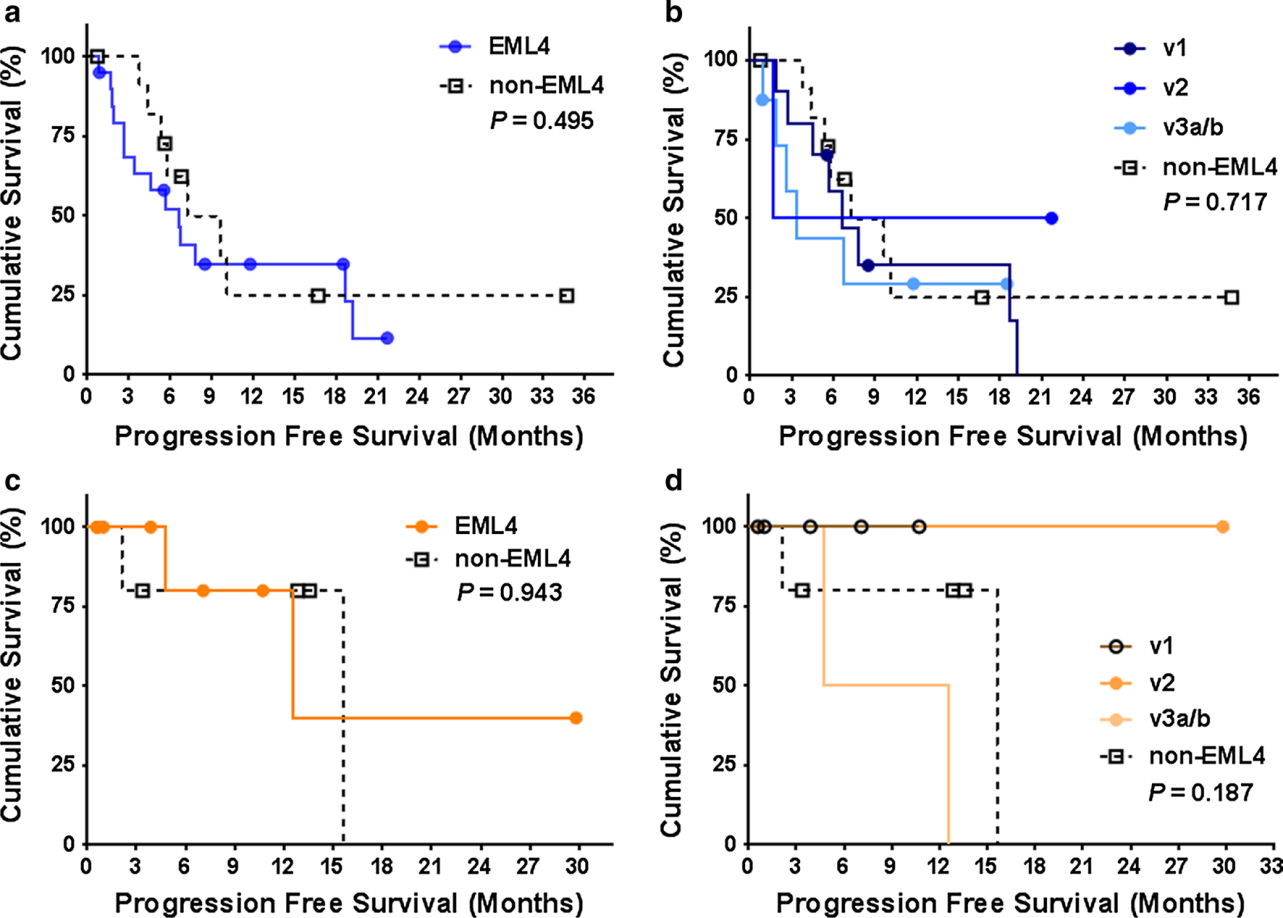
Kaplan–Meier curves of progression free survival of patients treated with crizotinib and ceritinib according to the *ALK* fusion variants. **a** *EML4* (*N* = 20) versus non-*EML4* (*N* = 12) on crizotinib. **b** Demonstration of progression free survival of each variant (v1, *N* = 10; v2, *N* = 2; v3a/b, *N* = 8; non-*EML4*, *N* = 12) on crizotinib. **c** *EML4* (*N* = 9) versus non-*EML4* (*N* = 5) on ceritinib. **d** Demonstration of progression free survival of each variant (v1, *N* = 5; v2, *N* = 1; v3a/b, *N* = 3; non-*EML4*, *N* = 5) on ceritinib. Each symbol on the plot marks a censored patient. v1, *EML4*-*ALK variant 1*; v2, *EML4*-*ALK variant 2*; v3a/b, *EML4*-*ALK variant 3a/b*

In patients who received first-line platinum-based chemotherapy, there was no significant difference in PFS according to *ALK* fusion variant (Fig. 2). With regard to pemetrexed, *EML4*-*ALK* fusion variants showed significantly superior PFS compared to non-*EML4* variants (Fig. 3a). When further analyzing the subtypes of *EML4*-*ALK* variants, v1 showed significantly better PFS than did the others (Fig. 3b). No significant difference according to *ALK* fusion variant was found in PFS of patients treated with crizotinib or ceritinib (Fig. 4). In the univariate Cox proportional hazards analyses for PFS on pemetrexed, v1 was determined to be a predictive factor for prolonged PFS, while non-*EML4* was identified as a risk factor for shorter PFS. However, in the multivariate analysis, v1 was the only significant predictive factor of longer PFS (Table 3).

**Table 3:** Cox proportional hazards regression analysis for progression free survival on pemetrexed

Variables	Univariate		Multivariate	
	HR (95 % CI)	*P* value	HR (95 % CI)	*P* value
*EML4*-*ALK variant 1*	0.262 (0.098–0.699)	*0.007*	0.262 (0.098–0.699)	*0.007*
*EML4*-*ALK variant 2*	2.364 (0.305–18.344)	0.410		
*EML4*-*ALK variant 3a/b*	1.366 (0.443–4.214)	0.587		
Non-EML4 variant	2.890 (1.191–7.013)	*0.019*		

Italic values indicate significance of *P* value (*P* < 0.05)

*HR* hazard ratio; *CI* confidence interval

## Discussion

In the present study, patients harboring the *EML4*-*ALK* variant, especially v1, had significantly prolonged PFS on pemetrexed therapy, whereas no difference in PFS was observed for those treated with ALK inhibitors, according to the *ALK* fusion variants. Since FISH was used as the gold standard method for enrollment in clinical trials of ALK inhibitors, information on *ALK* fusion variants was limited in previous studies, and the efficacy of chemotherapy or targeted therapy according to fusion variant was not established. In the present study, we confirmed the *ALK* rearrangements in patient with NSCLCs using FISH and IHC and additionally specified the *EML4*-*ALK* variants using RT-PCR, which identifies the largest number of *EML4*-*ALK* variants to date.

In the present study, *EML4*-*ALK* fusion v1 was the most common variant, identified in 38.5 % of all patients and accounting for 60.6 % of all *EML4*-*ALK* variants. This finding is consistent with previous studies [15, 16]. Non-*EML4* variants were the second most common, identified in 36.5 % of patients, which is slightly higher than that previously reported [3, 16]. Although the RT-PCR methods used in the present study were designed to detect 28 types of *EML4*-*ALK* rearrangements, only v1, v2, and v3a/b were identified in our patients. Among the *EML4*-*ALK* variants, v3a/b and v2 were the second and third most common types, as previously described [3, 15].

Most of the patients in the present study received ALK inhibitors as second- or further-line treatment, and the ORR to ALK inhibitors was far better than to platinum-based or pemetrexed chemotherapy. Crizotinib was used most commonly, followed by ceritinib and alectinib. Crizotinib was initially developed as a c-Met inhibitor, but was found to be an efficient inhibitor of *ALK* phosphorylation and signal transduction [17], and to be effective in the treatment of *ROS1*-rearranged NSCLCs [18, 19]. Ceritinib [20, 21] and alectinib [22, 23] are second-generation ALK inhibitors that can be used in patients with crizotinib resistance or intolerance. In the present study, the treatment response rate to ALK inhibitors was no different between the *EML4* group and non-*EML4* group, consistent with a previous study [3]. There was also no difference among *EML4*-*ALK* variants. Recently, during preparation of our manuscript, Yoshida et al. reported on the frequency of *ALK* fusion variants and the therapeutic efficacy of crizotinib according to the different variants in patients with *ALK*-rearranged NSCLCs; this approach was similar to that of our study [16]. They evaluated 35 patients with *ALK*-rearranged NSCLCs, and found that v1, the most common variant, was associated with superior PFS on crizotinib than non-v1 variants. This was not observed in our study. Further studies are required to investigate these discrepant findings. These two studies had similar limitations that could affect study results: both were retrospective studies with a small sample size. In addition, the treatment line for crizotinib differed across studies.

Although Yoshida et al. did not evaluate the therapeutic efficacy of second-generation ALK inhibitors such as ceritinib, we examined the therapeutic efficacy of ceritinib according to *ALK* fusion variants, and found no difference in the use of ceritinib as crizotinib. Notably, however, one patient with v2 variant achieved a CR on ceritinib treatment. The tumor of this patient exhibited an extensive papillary and micropapillary pattern with partly retained alveolar wall architecture, distinguishing it histologically from the usual pattern of *ALK*-rearranged tumors. In the present study, all v2 patients showed PR to crizotinib and CR to ceritinib. Moreover, both the ORR and DCR were 100 %, although v2 was found in only 3 patients—too small a number to draw a general conclusion. A previous in vitro study reported v2 as being the most sensitive to *ALK* inhibition, and explained that v2 has a shorter half-life compared with the other variants and is far more unstable since it has the longest N-terminus of *EML4* [24].

Pemetrexed, a folate antimetabolite that inhibits enzymes used in purine and pyrimidine synthesis, has been approved for malignant mesothelioma and NSCLC of non-squamous histology. Previous studies demonstrated an association between *ALK* rearrangement in NSCLC and prolonged PFS in patients treated with pemetrexed [25, 26]. The studies measured mRNA level of thymidylate synthase (TS), one of the catalytic enzymes thought to reduce sensitivity to pemetrexed, and showed a significantly lower TS mRNA level in *ALK*-rearranged tumor cells [26]. However, a more recent study with a larger cohort refuted these observations: it showed similar PFS of patients with *ALK*-rearranged and *ALK*-wild type NSCLCs [27]. They also measured mRNA level of TS and concluded that *ALK* rearrangement was associated with a lower TS mRNA level, but that PFS on pemetrexed treatment was not affected by *ALK* status [27]. So far, all previous studies on pemetrexed efficacy confirmed *ALK* rearrangement using the FISH method. Thus, frequency of *ALK* fusion variants in each study was unknown [25–27]. One possible scenario for the observed discrepancy regarding pemetrexed efficacy is that proportion of v1 might have differed in each previous study, because in the present study pemetrexed showed a significantly better PFS when used to treat v1 variants than when used to treat other variants. Although we could not examine the TS level in tumors because the remaining tumor tissue was not available due to previous extensive molecular examination, further validation is needed to clarify the mechanism of prolonged PFS of v1 on pemetrexed observed in the present study. Although crizotinib is the most efficient and verified target agent for patients with *ALK*-rearranged NSCLC, pemetrexed would be a good treatment option if patients harbor the v1 variant and cannot afford crizotinib. Subtyping *ALK* variants might predict the efficacy of pemetrexed.

## Conclusions

In conclusion, our study showed different PFS on pemetrexed treatment according to *ALK* fusion variant in lung adenocarcinoma. *EML4*-*ALK* variants, especially v1, had superior PFS than the other variants. We found no difference in PFS with ALK inhibitors according to *ALK* fusion variant. Further studies with large cohorts are required to confirm the different efficacy of pemetrexed or ALK inhibitors according to *ALK* fusion variants.

## Additional file

**Additional file 1: Table S1.** Metastatic sites according to *ALK* fusion variant. **Table S2.** Treatment history of patients before ALK inhibitors. **Fig. S1.** Histologic features of the tumor harboring *EML4-ALK variant 2.*

## Abbreviations

ALK: anaplastic lymphoma kinase
CR: complete response
Ct: threshold cycle
DCR: disease control rate
EGFR: epidermal growth factor receptor
EML4: echinoderm microtubule-associated protein-like 4
FFPE: formalin-fixed, paraffin-embedded
FISH: fluorescence in situ hybridization
IHC: immunohistochemistry
NSCLC: non-small cell lung cancer
ORR: objective response rate
PD: progressive disease
PFS: progression-free survival
PR: partial response
RT-PCR: real time-polymerase chain reaction
SD: stable disease
TKI: tyrosine kinase inhibitor
TS: thymidylate synthase
