# Supplementary material for: Clinical outcomes in ALK-rearranged lung adenocarcinomas according to ALK fusion variants
Source: J Transl Med. 2016 Oct 19;14:296. doi: 10.1186/s12967-016-1061-z (PMC5069800; doi:10.1186/s12967-016-1061-z)
Supplement: Supplementary file 1 — Additional file 1: Table S1. Metastatic sites according to ALK fusion variant. Table S2. Treatment history of patients before ALK inhibitors. Fig. S1. Histologic features of the tumor harboring EML4-ALK variant 2. [file 12967_2016_1061_MOESM1_ESM.docx]

**Supplementary Table 1.** Metastatic sites according to *ALK* fusion variant

|  | *EML4-ALK variant 1* (*N* = 20) | *EML4-ALK variant 2* (*N* = 3) | *EML4-ALK variant 3a/b* (*N* = 10) | Non-EML4 variants (*N* = 19) | Total (*N* = 52) | *P*-value |
| --- | --- | --- | --- | --- | --- | --- |
| Brain | 8 (40.0) | 2 (66.7) | 4 (40.0) | 9 (47.4) | 23 (44.2) | 0.873 |
| Liver | 4 (20.0) | 1 (33.3) | 2 (20.0) | 3 (15.8) | 10 (19.2) | 0.904 |
| Adrenal gland | 1 (5.0) | 1 (33.3) | 3 (30.0) | 1 (5.3) | 6 (11.5) | 0.092 |
| Lung/pleura | 13 (65.0) | 1 (33.3) | 8 (80.0) | 6 (31.6) | 28 (53.8) | **0.034** |
| Bone | 8 (40.0) | 0 (0.0) | 2 (20.0) | 6 (31.6) | 16 (30.8) | 0.924 |

**Supplementary Table 2.** Treatment history of patients before ALK inhibitors

|  | *EML4-ALK variant 1* (*N*=20) | *EML4-ALK variant 2* (*N* = 3) | *EML4-ALK variant 3a/b* (*N* = 10) | Non-EML4 variants (*N* = 19) | Total (*N* = 52) |
| --- | --- | --- | --- | --- | --- |
| **First line** |  |  |  |  |  |
| Platinum with pemetrexed | 5 (25.0) | 0 (0.0) | 6 (60.0) | 3 (15.8) | 14 (26.9) |
| Pemetrexed single | 0 (0.0) | 1 (33.3) | 0 (0.0) | 2 (10.5) | 3 (5.8) |
| Platinum without pemetrexed | 12 (60.0) | 1 (33.3) | 3 (30.0) | 10 (52.6) | 26 (50.0) |
| **Second line or beyond** |  |  |  |  |  |
| Pemetrexed single | 12 (60.0) | 0 (0.0) | 0 (0.0) | 6 (31.6) | 18 (34.6) |
| EGFR TKI | 6 (30.0) | 0 (0.0) | 2 (20.0) | 3 (15.8) | 11 (21.2) |
| Platinum without pemetrexed | 2 (10.0) | 0 (0.0) | 1 (10.0) | 1 (5.3) | 4 (7.7) |

TKI, tyrosine kinase inhibitor

**Supplement Figure 1.** Histologic features of the tumor harboring *EML4-ALK variant 2*

*
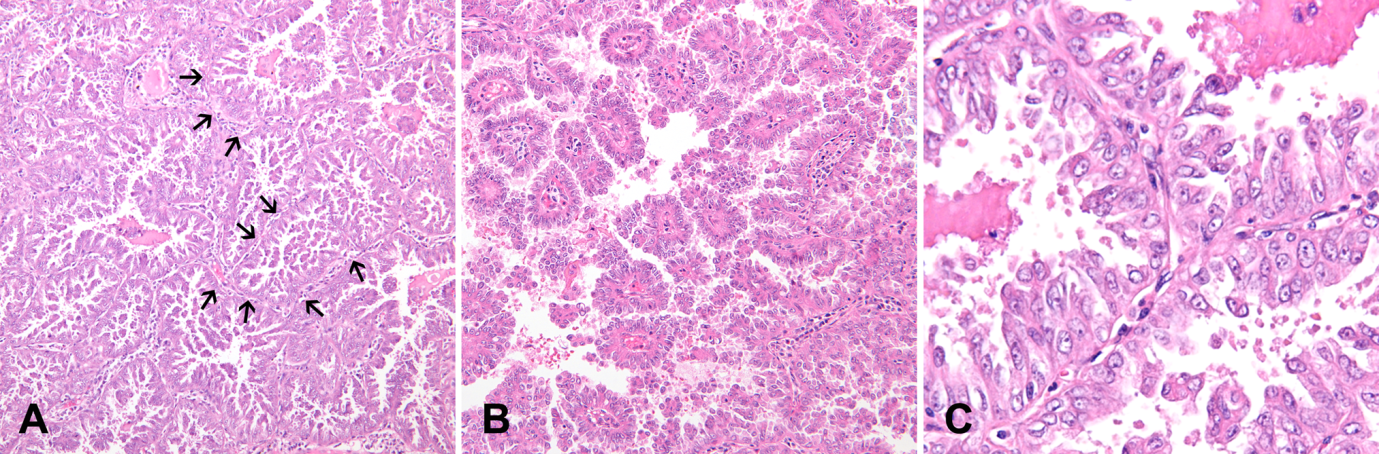
*

A 42-year-old female, who harbored v2, showed complete response (CR) to ceritinib. Six-months after lobectomy of the right lower lobe was performed to remove the 12 cm primary tumor (pathologic stage IIB; T3N0M0), the tumor recurred in the contralateral lung (rM1a). She received first-line pemetrexed monotherapy and showed stable disease for 6 months. Ceritinib was used for second-line therapy after progressive disease on pemetrexed, and CR was achieved for 29.8 months.

(A) At low power magnification, partly retained alveolar walls (arrows) are noted with prominent floating papillary and micropapillary tumor clusters. A cribriform or solid pattern is absent.

(B) Papillary tumor clusters having fibrovascular cores are admixed with micropapillary clusters and singly scattered tumor cells devoid of a fibrovascular core.

(C) Most tumor cells show nuclear clearing with prominent nucleoli. Note the hobnail pattern resulting from long cytoplasmic process of tumor cells attached to the alveolar walls.
